# i-Assess: Evaluating the impact of electronic data capture for OSCE

**DOI:** 10.1007/s40037-018-0410-4

**Published:** 2018-02-27

**Authors:** Sandra Monteiro, Debra Sibbald, Karen Coetzee

**Affiliations:** 1 Department of Health Research Methods, Evidence and Impact, McMaster University, Hamilton, Canada; 2 Touchstone Institute, Toronto, Canada; 3 Department of Pharmacy, University of Toronto, Toronto, Canada

**Keywords:** Tablet based assessment, OSCE, Psychometrics, Generalizability

## Abstract

**Introduction:**

Tablet-based assessments offer benefits over scannable-paper assessments; however, there is little known about the impact to the variability of assessment scores.

**Methods:**

Two studies were conducted to evaluate changes in rating technology. Rating modality (paper vs tablets) was manipulated between candidates (Study 1) and within candidates (Study 2). Average scores were analyzed using repeated measures ANOVA, Cronbach’s alpha and generalizability theory. Post-hoc analyses included a Rasch analysis and McDonald’s omega.

**Results:**

Study 1 revealed a main effect of modality (*F* (1,152) = 25.06, *p* < 0.01). Average tablet-based scores were higher, (3.39/5, 95% CI = 3.28 to 3.51), compared with average scan-sheet scores (3.00/5, 95% CI = 2.90 to 3.11). Study 2 also revealed a main effect of modality (*F* (1, 88) = 15.64, *p* < 0.01), however, the difference was reduced to 2% with higher scan-sheet scores (3.36, 95% CI = 3.30 to 3.42) compared with tablet scores (3.27, 95% CI = 3.21 to 3.33). Internal consistency (alpha and omega) remained high (>0.8) and inter-station reliability remained constant (0.3). Rasch analyses showed no relationship between station difficulty and rating modality.

**Discussion:**

Analyses of average scores may be misleading without an understanding of internal consistency and overall reliability of scores. Although updating to tablet-based forms did not result in systematic variations in scores, routine analyses ensured accurate interpretation of the variability of assessment scores.

**Conclusion:**

This study demonstrates the importance of ongoing program evaluation and data analysis.

## What this paper adds

Changes in rater-based assessment processes may have unintentional effects on overall scoring patterns. There are limited examples of comprehensive statistical analyses of assessment data for evaluating changes in scoring process. In particular, none focus on the impact of technology on scoring variability. Changes in the format of the scoring system may influence the variability of ratings in high-stakes contexts. In isolation, small variations may be interpreted as significant in the context of evaluating the reliability of ratings. However, comprehensive investigative and statistical methods offer a more accurate perspective.

## Introduction

Rater-based assessments are considered sensitive to many factors that can influence the overall range of scores and the amount of error or variance. The rater cognition literature points to factors such as mental effort [1], first impressions [2], social judgments [3], task specificity [4] and individual differences [5, 6]. Mental effort in particular has been explored within the varying complexity of the rating task. Notably, inter-rater reliability has been shown to be higher when task demands are lower and less complex [1].

Task demands result from many factors, both relevant and irrelevant to the task of assessing performance. Factors related to irrelevant task demands include the visual format of the rating form [7–9] and relevant task demands may result from the number of competencies or items being evaluated [1]. That is, although raters should be focused on the candidate’s performance, they may be distracted by the challenge of understanding their rating form or attending to multiple behaviours at once. As changes to both relevant and irrelevant rater-based processes may influence the reliability of scores, it is critical to evaluate the role of assessment format on scores, especially in high-stakes assessments. These concerns are particularly relevant to the Objective Structured Clinical Exam (OSCE), which is an intricate interplay of candidate performance, rater cognition and assessment format [10, 11].

Typical OSCEs contain multiple stations assessing various areas of professional practice [11]. The OSCE design is the result of decades of development and there exists strong psychometric and qualitative evidence regarding its acceptability, defensibility, validity and reliability in many domains [10–14]. A standard OSCE is used to assess professional competencies, or the application of knowledge to a variety of complex scenarios. For example, an OSCE to assess competencies in nursing might include a station that requires the candidate to communicate a post-surgical management plan to an elderly patient. While the OSCE has been reviewed extensively in the literature, changes to any aspects of the exam design may yet influence aspects of utility, namely the reliability or generalizability of scores [10–14].

One contextual change that is likely in many health professions assessment centres is a transition from paper-based assessments to tablet-based assessments [15–18]. As is common for OSCEs, raters currently use optical scan sheets to enter their ratings for each candidate. These sheets are then scanned by a computer to transfer data from paper to electronic format. This was not always standard practice, however, as it was not too long ago that assessment centres switched from entering data by hand [17]. Although optical scan sheets are now seen as more efficient compared with previous methods, for very large assessments they can be problematic [16].

The process for ensuring data security and integrity includes creating unique barcode labels for each candidate and having runners check each scan sheet for completeness. These processes can be time consuming and expensive and are still not error free. There is inevitably a loss of data as raters sometimes do not complete their ratings, for a variety of reasons [13, 16]. For example, it is not unheard of for raters to fall asleep, lose focus or move on to the next candidate without completing their assessment. These lapses may be seen as costly in the assessment process, leading to critical loss of data and reduced accuracy.

Technology-driven changes to rating processes can make assessments more efficient and cost effective, but may have unintended consequences on the variance of scores. For example, task irrelevant perceptual differences between paper and tablet-based assessments may influence overall task demands or change how raters orient to the rating scale [7–9]. Prior studies that have examined the transition from paper to tablet in the OSCE have focused on the rater’s perceptions of the process [16] and the influence on the quality of raters’ written comments [15]. The current study was aimed at evaluating the influence on the quality of the assessment scores, particularly the variance and generalizability of scores.

While specific issues or challenges may differ by assessment context, the current study explored a very common design utilized within high-stakes OSCEs and may be enlightening to others considering technological changes as well. This study also incorporated several statistical analyses ideal for the OSCE context and may be enlightening regarding conceptual challenges in the development of measurement tools.

## Research goals

The current study was developed to understand the local impact of introducing tablet-based OSCE assessments in a specific context at Touchstone Institute in Toronto, Canada. The study was designed to examine whether there would be any systematic changes in average assessment scores or variance of scores, as a direct result of introducing tablets in the assessment context.

### Assessment context

Touchstone Institute in Toronto, Canada is a competency evaluation and assessment centre for internationally trained health professionals. Most notably, Touchstone Institute plays an integral part in evaluations of internationally trained physicians and nurses and these evaluations include performance assessments in an OSCE.

The internationally educated nurse competency assessment program (IENCAP) includes a 12 Station OSCE. There are typically between 100 and 200 nurse candidates registered for a single administration, so they are assigned to different circuits across concurrent tracks in one day. Typically, this is also spread over multiple sessions.

At any given station, candidates interact with standardized clients and the rater in the room. Throughout each 10-minute station, raters evaluate individuals on a set of 10 items (i. e. competencies). Some of the items are behavioural indicators focused on the client encounter and some are communicative competencies elicited during a 2-minute oral exam at the end of the encounter with the standardized client. For the purpose of this study, a *competency score* is a single rating of one item in one station. All competencies are rated on a 5-point global rating scale. A *station score* is the average of all competency scores evaluated in that station. A *total score* is the average of all station scores.

Each rater is selected by the College of Nurses of Ontario, from a pool of qualified nurses representing subject matter experts. The raters are currently in practice, and experienced in writing for and examining the IENCAP over multiple administrations. All raters are provided with an orientation session and five hours of specialized training.

For the purpose of this study, all nurse raters will be referred to as raters, and all nurse candidates will be referred to as candidates.

## Methods

### Assessment software development

**Fig. 1:**
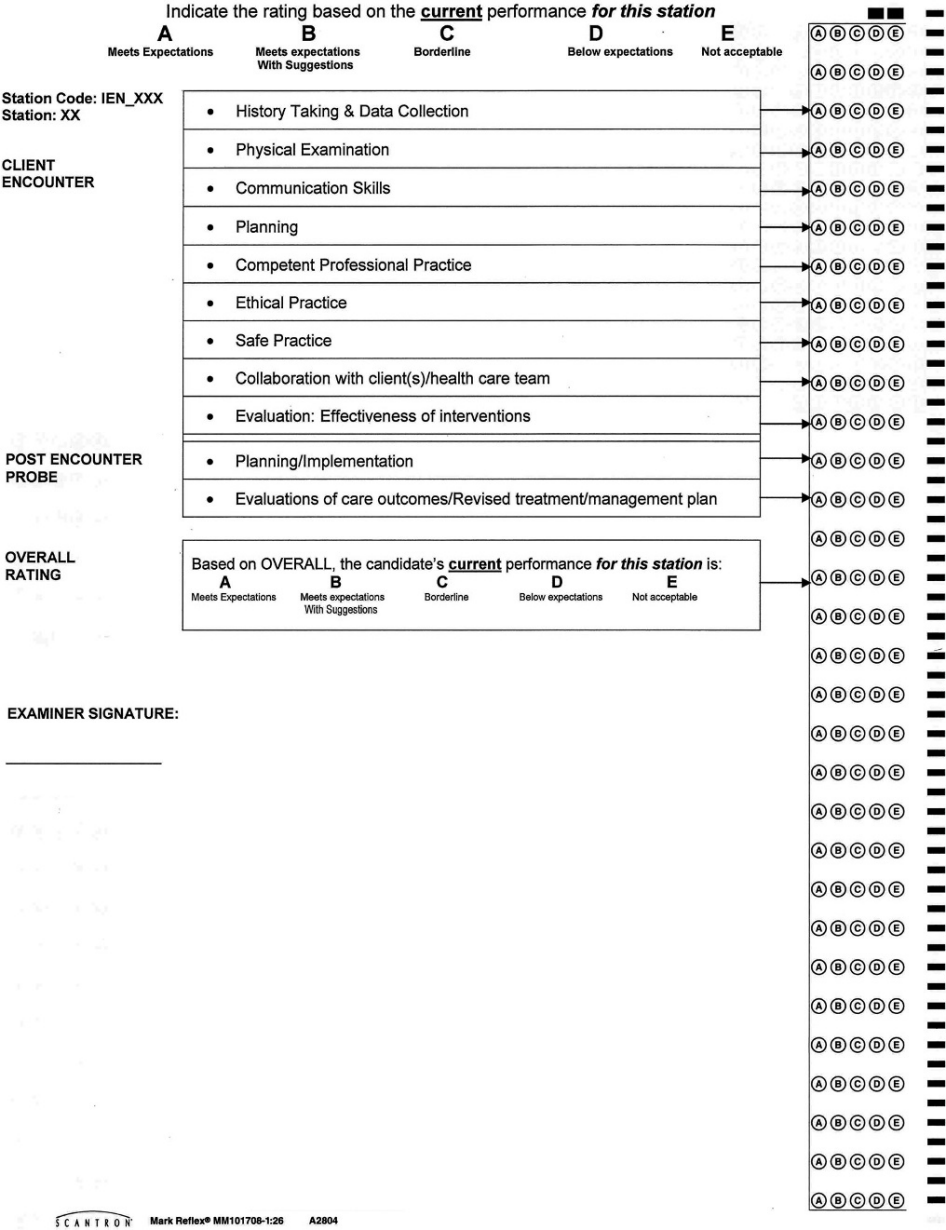
Image of scannable sheet typically used by Touchstone Institute. Performance rating anchors are described at the top of the page and raters are instructed to select the corresponding letter for each competency assessed. In this test sheet, 11 competencies are shown. In this study scores for 10 competencies are analyzed as Physical Examination is not evaluated in all stations

The optical scan sheets used by Touchstone Institute employ a global rating scale displayed horizontally with the highest score on the left; the letter A represents the highest score possible (see Fig. 1 for an example). The rating scale ranges from A (Meets Expectations) to E (Not Acceptable), and raters are provided with print outs describing the anchors and the unique behavioural and competency requirements for each performance level at each station.

**Fig. 2:**
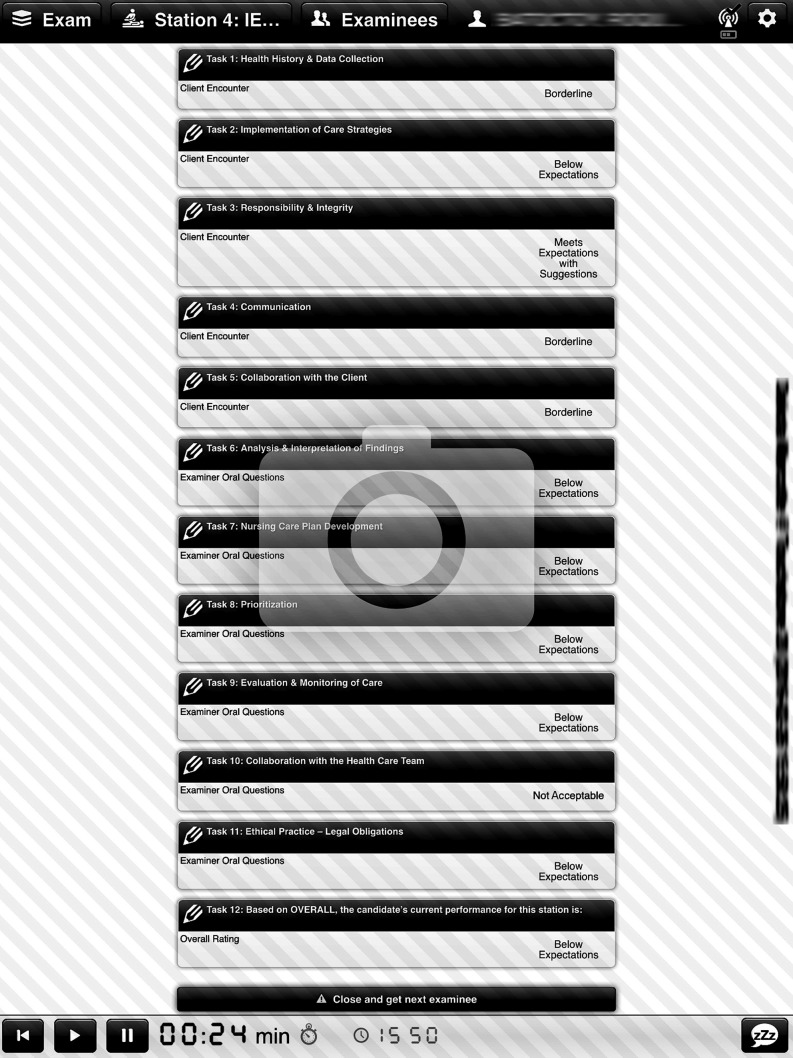
Screenshot of current tablet interface for raters. This image shows a hypothetical candidate receiving scores such as ‘Borderline’ or ‘Not Acceptable’ for 11 competencies. Similar to the scan test data, only scores for 10 competencies were analyzed in the current study

In 2015, Touchstone Institute introduced a tablet-based rating system in collaboration with UCAN®. Consultations between the exam design teams from Touchstone Institute and UCAN® led to the development of a tablet interface with larger font, additional colour and drop down menus of vertical global rating scales with the highest score on top (see Fig. 2 for a screen capture as example). The shift from a horizontal to a vertical scale alone can influence the way in which raters rely on anchors [7–9]. Moreover, the anchors are only visible when the drop down menu is accessed on the tablet, in contrast to the scan-sheet format, which displays the anchors at the top of the page. Furthermore, in the scan-sheet format, raters are able to detect a visual pattern of all scores they have entered for a candidate at once. Potentially, this can lead to pattern following or an anchoring bias to the extremes of the scale [19]. This difference may also lead to modulations of mental effort, either reducing or increasing the complexity of the task for the rater [1]. Additionally, with scan-sheet scoring, raters are free to return to one or more ratings from a previous candidate and rescore at any time up until the scan sheet is collected by staff (generally every three rounds but sometimes longer). This is not possible within the tablet interface which will not allow a rater to proceed without completing the rating form; as a result, there may be higher or lower consistency between scores.

To evaluate these changes and potential consequences, two studies were conducted. In particular assessment scores were analyzed to determine if the transition to tablet-based assessment lead to systematic changes in the variance of scores, internal consistency within stations, or the overall generalizability of scores. Generalizability theory and general linear modelling informed the analyses, but logistics informed the design, as we only had enough tablets for roughly half our candidates at any one administration.

### Procedure

Tablet-based assessments were first piloted in three separate administrations (i. e. different exam dates) to evaluate any logistic or data security issues. Tablets were assigned to non-critical raters in viewing rooms to test the operations and usability. The scores taken from the viewing room raters in the pilot studies did not count towards exam results and we do not report or analyze those here. Indeed, psychometric analyses of these pilot data were inconclusive. Instead we relied on those raters to provide qualitative feedback. As the operations were deemed acceptable based on raters’ feedback, we proceeded to a staged implementation in two administrations of the IENCAP in 2015. These two administrations were the first time tablet-based assessments were collected for high-stakes purposes at Touchstone Institute.

Each administration of the IENCAP followed the same exam blueprint (i. e. covered the same broader competency domains) but included different sets of stations and station scripts. That is, for each station, the same 10 individual competencies or behavioural and communicative objectives were assessed; however, they were defined to match the station context. Additionally, across administrations, station order would differ. For example, station 1 may have dealt with mental health issues in one administration but chest pain in another. For stations that addressed the same issue, such as chest pain, there may be slight differences in features across administrations, such as patient characteristics, scripts or prompts. For all these reasons, station scores from different administrations could not be combined in one analysis. This led to two separate study designs for two different exam administrations. Using generalizability theory, however, does facilitate comparisons of variance across different studies and contexts.

### Study design 1

In the May administration (*n* = 154), the IENCAP consisted of 6 tracks with the identical 12-station circuit in each. Half the tracks were assigned to be evaluated using tablets and half were assigned to standard paper optical scan sheets. This was a between-subjects design, comparing tablet assessment in one group of candidates and scan-sheet assessment for another group of candidates.

### Study design 2

In the August administration (*n* = 90), there were again 6 tracks, and a different set of 12 stations. In this design, all candidates were evaluated using both tablets and paper in a counterbalanced design. On three tracks, raters were assigned tablets in the odd numbered stations and scan sheets on the even numbered stations. For the other 3 tracks, this was reversed. This study was a within-subject design, comparing tablet assessments on half the stations to paper assessments on the other half.

### Control study

For comparison purposes, we made a post-hoc decision to include a third set of different stations in the current study. An analysis of the prior January administration (*n* = 116) was included as it was one of the last exams scored entirely using scan sheets. Similarly, to May and August, the January exam consisted of 12 stations across 6 tracks.

Unfortunately, the exam blueprint changed at the same time that tablets were implemented across an entire administration. Therefore it was deemed not appropriate to include data from an exam scored entirely by tablets.

### Ethical considerations

The lead author is faculty at McMaster University and requested approval from the Hamilton Integrated Research Ethics Board, which determined this study was exempt from ethics review as it was a quality evaluation and improvement study. Additionally, as part of the quality assurance and intake process, candidates are presented with a consent form. They are asked to consent to allow Touchstone Institute to record and access their scores for the purposes of evaluation, quality assurance and quality improvement. They are explicitly informed that aggregate data may be shared in order to evaluate the assessment process. Touchstone Institute has a policy in place to store assessment data indefinitely for the purposes of program evaluation.

### Analysis

The current study employed both general linear modelling (analysis of variance—ANOVA) and generalizability theory. Although generalizability theory is less commonly known, we describe only the specific methodology used here. For a more theoretical foundation for the analyses describe please refer to the work of Brennan [20] and Streiner & Norman [21]. In previous work, we demonstrated that scores within IENCAP OSCE stations appeared unidimensional, while scores of the same competencies appeared multidimensional across stations [22]. This finding indicates that Cronbach’s alpha is an appropriate measure of internal consistency within station; however, for comparison purposes, we report both Cronbach’s alpha and McDonald’s omega [23]. Finally, because of the high internal consistency within station, it was deemed appropriate to submit average station scores to the ANOVA and *g*-studies. This decision builds on previous work showing no meaningful impact on reliability as a result of analyzing individual item scores compared with the case average in key features exams [24, 25]. However, for comparison purposes we report the absolute *g*-coefficients when submitting all 10 individual competency scores and when only submitting the average station score.

### Relationship between modality and variance of station scores

Tab. 1 summarizes aspects of study designs, the analyses and main results. In study design 1 with 154 candidates, the 12 station scores were submitted to a repeated measures ANOVA with one within subject factor of station at 12 levels and one between-subject factor of rating modality at two levels (tablet or scan sheet). The influence of individual tracks was not evaluated.

**Table 1:** Summary of the study designs, analyses and main results. Overall, there were 3 facets: station, modality and candidate. Candidate was always the facet of differentiation

	Study 1 May	Study 2a August	Study 2b August	Control January
*N*	154	46	44	116
Mean total score on a 5-point scale (SD)	3.19 (0.52)	3.40 (0.46)	3.48 (0.42)	3.19 (0.47)
Change in average scores	0.4 Tablet scores were 8% lower on average	0.1 Tablet scores were 2% lower on average		–
Reliability—Cronbach’s alpha (confidence interval)	0.88 (0.85 to 0.91)	0.82 (0.75 to 0.89)	0.84 (0.78 to 0.91)	0.86 (0.83 to 0.90)
Reliability—McDonald’s omega (confidence interval)	0.88 (0.85 to 0.91)	0.82 (0.74 to 0.90)	0.84 (0.78 to 0.91)	0.87 (0.83 to 0.90)
Internal consistency of 12 stations—McDonald’s omega	0.91 to 0.96	0.89 to 0.98	0.88 to 0.97	0.90 to 0.96
G-Study design	2 facets crossed, candidate nested	2 facets crossed, station nested	2 facets crossed, station nested	2 facets crossed
*Variance components*				
Facets				
– Station (Random)	0.03	0.04	0.05	0.05
– Station nested in Modality	–	0.22	0.03	–
– Candidate (Differentiation)	–	0.17	0.14	0.20
– Candidate nested in Modality	0.20	–	–	–
– Modality (Random)	0.07	0.02	0.0001	–
– Competency	0.02	0.01	0.01	0.02
– Residual	0.35	0.36	0.32	0.34
Absolute g‑coefficient for 10 competency scores as repeated measures across 12 stations (Index of dependability)	0.65	0.73	0.75	0.83
Absolute g‑coefficient using 12 station means (Index of dependability)	0.66	0.74	0.81	0.85
Inter-station generalizability	0.3	0.3	0.3	
Inter-modality generalizability	–	0.8		–

In study design 2, both counterbalanced groups (*n* = 46 and 44) were analyzed separately. The 12 station scores were submitted to a repeated measures ANOVA with two within subjects factors (modality at two levels and station at 6 levels).

For comparison, the 12 station scores for the control group (January exam, *n* = 116) were also analyzed using a repeated measures ANOVA with one within subjects factor of station at 12 levels.

### Relationship between modality and internal consistency of station scores

Standard quality assurance practice within Touchstone Institute is to report Cronbach’s alpha as a measure of internal consistency for scores within each station. Because of recent criticisms of the use of this metric, we also calculated McDonald’s omega [23]. A descriptive analysis of coefficients was conducted to determine if there were meaningful changes in internal consistency when assessments were recorded by tablet compared with scan sheets.

### Relationship between modality and generalizability of station scores

Average station scores from each administration were submitted to generalizability studies using G_String version 6.3.8 which runs on the uR Genova program. G_String was developed and coded by Ralph Bloch, with assistance from Geoff Norman, formerly Assistant Dean of the Program for Educational Research and Development, Faculty of Health Sciences, McMaster University. G_String relies heavily on Brennan’s formulation [20].

The primary question was to determine if there was a different generalizability coefficient when candidates were assessed using tablets or scan sheets. The question addressed: Was the change in assessment modality reflected in a change in the absolute *g*-coefficient [20, 21]? The absolute *g* was selected as a representation of an index of dependability and as a conservative estimate of how well scores from one context in the exam can generalize to any other context, either within the same exam or a different one.

In the analysis of the January administration, there were two crossed facets. The facet of differentiation was candidate, and station was a random facet.

In the analysis of the May administration, 72 candidates were nested in tablet assessment and 82 were nested in scan-sheet assessment. Within each nested condition, there were two crossed facets: candidates (facet of differentiation) and station (random facet).

In the analysis of the August administration, a separate *g*-study was conducted for each of the two counterbalanced groups (*n* = 46 and *n* = 44). Six stations were nested in each modality. In each modality there were two facets: Candidates (facet of differentiation) and station (random facet) were crossed.

## Results

### Qualitative and process related findings

#### Rater feedback

Since the introduction of tablet-based marking in 2015 the raters have been positive towards the new technology. Technological issues have been minimal and non-critical.

#### Influence of tablet assessment on administrative issues

The incorporation of tablet technology helped to reduce the amount of paper used, and the amount of time it takes to prepare for an assessment. The operations of assessments are now more cost-effective and eco-friendly. Previously a Scantron® test sheet would have been created for every candidate for each station (an estimated 12 stations X approximately 150 candidates).

Typically, the manufacturing, production and quality assurance process for scan test sheets prior to an administration took approximately 3–4 weeks’ time. The tablet OSCE system reduced this amount of time to 1 week. Using scan sheets, the scanning and cleaning process delayed the availability of candidate scores for up to 3 weeks. The tablet OSCE system now allows access to the candidate scores immediately post-exam.

Previously, with the use of scan-test sheets, a staff member was appointed to each track on exam day to ensure that each test sheet had been used appropriately (with the candidate barcode attached, only pencil used, filled a bubble for every competency listed, did not fill in any extra bubbles, completed signature section, etc.). The need for this position is now eliminated, as the tablet will not allow an incomplete test form to be submitted, reducing the margin of error. There is no longer a possibility of a rater accidentally failing to grade a competency for any candidate as rater behaviour can be monitored in real time through the tablet system. In previous administrations, an error rate of between 2 to 5% was expected due to missing scores or errors in data input or scanning. For candidates assessed using tablets, there were no missing scores.

## Quantitative psychometric findings

### The relationship between modality and variance of scores

Study design 1 (the May exam) revealed a main effect of modality (*F* (1,152) = 25.06, *p* < 0.001). There was also a main effect of station (*F* (11, 1,672) = 30.88, *p* < 0.001) and an interaction (*F* (11, 1,672) = 19.06, *p* < 0.001). The average tablet-assessed total score was 3.39 (95% CI = 3.28 to 3.51), a 10% increase over the average scan-sheet total score of 3.00 (95% CI = 2.90 to 3.11). As a comparative analysis, we re-submitted the 10 raw competency scores per station into the repeated measures ANOVA, and one between-subjects factor of modality. The results were qualitatively identical with a main effect of modality (*F* (1, 149) = 25.72, *p* < 0.001). The main effect of station was also similar (*F* (11, 1,639) = 30.96, *p* < 0.001) as was the interaction (*F* (11, 1,639) = 18.3, *p* < 0.001). The difference between scores for tablet, 3.41 (95% CI = 3.3 to 3.52) and scan sheet, 3.01 (95% CI = 2.91 to 3.12) remained unchanged. This was to be expected given the strong correlation between competency scores within each station [22].

In study design 2a, modality was again a main effect (*F* (1, 45) = 9.38, *p* < 0.01) as was station (*F* (5, 225) = 16.85, *p* < 0.001) and there was a significant interaction between modality and station (*F* (5, 225) = 23.41, *p* < 0.001). In study design 2b, modality was a main effect (*F* (1, 43) = 6.44, *p* < 0.01) as was station (*F* (5, 215) = 14.59, *p* < 0.001) and there was a significant interaction (*F* (5, 215) = 5.34, *p* < 0.001).

However, the overall difference between scan sheet and tablet scores was meaningfully reduced to 2% and the direction of the difference changed as average scan-sheet scores were higher (3.36, 95% CI = 3.30 to 3.42) than average tablet scores (3.27, 95% CI = 3.21 to 3.33). This was not considered a meaningful difference.

### Did the rating modality influence the internal consistency within stations?

The internal consistency (alpha and omega) of all 12 stations was ≥0.8 in all 3 exams, regardless of whether the modality was paper or tablet. Anecdotally, this is consistent with all previous exams that were assessed using scan sheets only.

### Was the generalizability of scores different for tablet or scan sheet assessments?

The inter-modality reliability for the August administration was 0.8, suggesting strong agreement between assessments recorded on either modality. A conservative index of dependability of the January exam (*n* = 116), which was scored on scan sheets only, was 0.83. However, the index of dependability of the May exam was lower at 0.65. The two August studies were in the middle with dependability indices of 0.73 and 0.75.

The inter-station reliability, or ability to generalize from one station to another station, regardless of modality, was unchanged at 0.3 in the August exam (*n* = 46 and *n* = 44), the January exam (*n* = 116) and the May exam (*n* = 154). These findings indicate a highly context-dependent impact of station.

### Post-hoc Rasch analysis

**Fig. 3:**
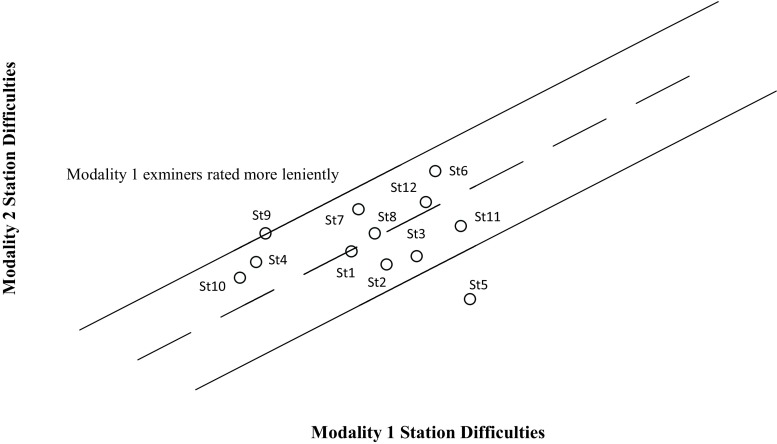
Twelve Rasch calculated station difficulties compared for Modality 1 and Modality 2. This scatterplot indicates that scores for the 12 stations were similar in difficulty for both modality 1 and 2 (i. e. tablet and scan sheet)

To examine the possibility that raters assigned to use tablets to record assessment scores in the May exam were generally more lenient, we conducted a Rasch analysis of examiner rating patterns using Facets software. Two sets of station difficulty values based on the paper and tablet modality scores were calculated and compared. A difference of 0.50 logit units or more between the two difficulty values for each station was used to determine whether or not examiners assigning scores using either the paper or tablet modality behaved more leniently or stringently in their score allocation. The difficulty scores for each station/modality are plotted in Fig. 3, which indicates no relationship between modality and station difficulty.

## Discussion

### Summary of results

The current study evaluated the psychometric properties of a high-stakes OSCE to determine if changing the rating modality was related to any subsequent systematic changes in score patterns or variance; no systematic changes were found. Scores were examined specifically for variance and generalizability, which remained fairly stable across 3 out of 4 studies. The exception was the May exam which revealed small differences between tablet and scan-sheet average scores and a drop in the index of dependability. Extensive analyses were conducted, however the results do not offer any explanations for these initial findings and we interpreted them as specious. First, internal consistency for stations did not change in any meaningful way. This suggests that the change to tablet-based scoring did not interfere with the relationship between scores. Second, overall exam reliability was stable using either Cronbach’s alpha or McDonald’s omega. This suggests that assessments of candidate performance remained stable across all stations. Third, there were no further fluctuations in scores or differences between modalities in subsequent assessments. This suggests a potential Type 1 error in the analysis of the May exam. This was supported by a Rasch analysis of station difficulties indicating no difference between tracks assessed using tablets and those assessed using scan sheets.

The attraction of technology to support more efficient administration is likely to lead to increased spread over time and the current study did not find evidence that implementation of tablet-based assessments would compromise the quality of the exam in our context. This study is a first psychometric examination of the relationship between exam quality and technology changes. Although an ANOVA, which is based in classical test theory, revealed a difference between modalities in one exam, a Rasch analysis did not support this finding. However, these findings emphasize the importance for ongoing quality assurance to distinguish between stable and specious psychometric effects. This study may also serve as a resource for similar studies in the future.

### Theoretical implications and future directions

Advances in technology offer greater efficiency within assessment [11]. However, when implementing changes in rater-based assessments, it is necessary to ensure that changes in process have not had unintentional effects on overall scoring patterns. Studies of rater cognition, for example, would suggest that changes in assessment format might influence scores. While it was not the goal of the current study to evaluate rater cognition directly, it was theorized that changes in the rating format or interface would have an influence on rater cognition that could lead to changes in the reliability of the exam. It is possible that changes in how raters interacted with the assessment tool affected the variance of scores in Study 1 as the index of dependability was lower at 0.66. There were also small differences between sub-groups in the August exam; 0.74 compared with 0.81. Additional analyses however did not support a conclusion that tablet-based assessments were less reliable.

Studies on scale development also suggest that a change in assessment tools may influence how raters assigned scores. In clinical studies of postoperative pain, the orientation of the rating scale used has been shown to influence how patients evaluated their level of pain and the frequency of extremely low scores (e. g. scores of zero; 7–9). In one study, pain scores were found to have higher sensitivity with horizontal scales than vertically oriented scales. These findings have serious implications for the assessment of clinical competence if a candidate’s score can be influenced by the orientation of a rating scale. Additionally, it is well established that anchoring is possible with rater-based assessments [17] and it is unclear if the orientation of a rating scale (i. e. vertical or horizontal) will have any influence on performance assessments. Within the current study scale orientation was embedded within modality so it was not possible to evaluate the influence of orientation independently of other factors. The potential for anchoring patterns that depend on the orientation of the scale should be part of future design considerations.

The analyses conducted were informed by expert knowledge of the patterns of data as well as theories of measurement. At Touchstone Institute, we have previously demonstrated a strong relationship between competency scores that is unified by station context [22]. This knowledge informed our approach to analysis. It is critical to understand the relationship between assessment scores before using them to make high-stakes decisions, and similarly before evaluating the impact of process changes. The unique context of this study also contributes to an understanding of the complexities of implementation of tablet scoring and how designs aimed at supporting principles of assessment in our context might inform practice or be transferable in enhancing development across broader contexts. It is hoped that this research will stimulate continued study of the features and principles explored. The approach used in this study may also be valuable for routine quality assurance processes when evaluating changes to assessment designs.

### Limitations

This study is limited by the unique context. This study does not preclude different effects occurring with other changes in context, or technology. As well, this is only one study and assessment centres considering the adoption of electronic platforms for OSCEs should conduct their own quality assurance studies.

## Conclusion

Updating to computer-based forms may affect variance in OSCE station scores; however, we noted no consistent systematic bias. Importantly to our quality assurance process, there was no meaningful change in overall reliability. These results were sufficient to suggest that full transition to tablet-based assessment would not compromise our standards for assessment.
